# Terpenoid-Rich Extract of *Dillenia indica* L. Bark Displays Antidiabetic Action in Insulin-Resistant C2C12 Cells and STZ-Induced Diabetic Mice by Attenuation of Oxidative Stress

**DOI:** 10.3390/antiox11071227

**Published:** 2022-06-23

**Authors:** Bo-Rim Song, Md Badrul Alam, Sang-Han Lee

**Affiliations:** 1 Department of Food Science and Biotechnology, Graduate School, Kyungpook National University, Daegu 41566, Korea; sbr9707@knu.ac.kr (B.-R.S.); mbalam@knu.ac.kr (M.B.A.); 2 Food and Bio-Industry Research Institute, Inner Beauty/Antiaging Center, Kyungpook National University, Daegu 41566, Korea

**Keywords:** *Dillenia indica* L. bark, GLUT4, Akt, ROS, Nrf2, HO-1

## Abstract

Insulin resistance (IR) plays a key role in the pathogenesis and clinical outcome of patients with multiple diseases and diabetes. In this study, we examined the antidiabetic effects of a terpenoid-rich extract from *Dillenia indica* L. bark (TRDI) in palmitic acid-induced insulin resistance (PA-IR) in C2C12 myotube and a streptozotocin (STZ)-induced diabetic mice model and explored the possible underlying mechanism. TRDI showed potential DPPH- and ABTS-radical scavenging effects with a half-maximal inhibitory concentration (IC_50_) value of 9.76 ± 0.50 µg/mL and 17.47 ± 1.31 µg/mL, respectively. Furthermore, TRDI strongly mitigated α-glucosidase activity with an IC_50_ value of 3.03 ± 1.01 µg/mL, which was 92-fold higher than the positive control, acarbose (IC_50_ = 279.49 ± µg/mL). TRDI stimulated the insulin receptor substrarte-1 (INS-1), downregulated phosphoinositide-dependent kinase-1 (PDK1) and protein kinase B (Akt) in both normal and PA-IR C2C12 cells as well as in STZ-induced diabetic mice, enhanced glucose transporter 4 (GLUT4) translocation to the plasma membrane (PM), and increased glucose absorption. Furthermore, TRDI administration significantly reduced PA-induced reactive oxygen species (ROS) formation in C2C12 cells and increased the protein level of numerous antioxidant enzymes such as superoxide dismutase 1 (SOD1), catalase (CAT), glutathione peroxidase-1 (GPx-1) and thioredoxin reductase (TrxR) both in vitro and in vivo. Furthermore, TRDI facilitated nuclear factor erythroid 2 related factor 2 (Nrf2) nuclear translocation and increased HO-1 expression in PA-IR C2C12 cells and STZ-induced diabetic mice. However, for the inhibition of Nrf2, TRDI failed to resist the effects of IR. Thus, this study provides new evidence to support the use of TRDI for diabetes treatment.

## 1. Introduction

Diabetes is a widespread global health issue characterized by elevated blood glucose levels, which are associated with high mortality and morbidity rates, and a variety of disease-specific side effects and complications, including neuropathy, cardiovascular disease, weakness, high blood pressure, nephropathy, retinopathy, and other ailments [1]. Globally by 2035, the number of diabetic patients is predicted to reach 592 million [2]. Type 1 diabetes (T1DM), type 2 diabetes (T2DM), gestational diabetes, and other diabetes types are categorized by the American Diabetes Association [3]. T2DM is caused by a combination of decreased tissue insulin sensitivity, insulin resistance (IR), and pancreatic-cell failure [4]. IR is critical for T2DM development and is caused by decreased glucose absorption into adipose tissue and skeletal muscle. Increasing evidence now suggests that excessive plasma free fatty acid (FFA) concentrations may be the primary cause of IR [5]. Palmitic acid (PA) is one of the most prevalent FFAs, accounting for approximately 30–35% of total FFAs in human plasma, and directly affects insulin signaling in cultured hepatocytes and myotubes [6]. FFAs have a variety of effects on insulin action and glucose metabolism. The liver and skeletal muscle are the sites of the most significant interactions. In hepatocytes, a high FFA content inhibits insulin action. Glucose and FFAs compete with one another as energy sources in skeletal muscle. Muscle cells prefer to oxidize FFA over glucose when the FFA level is high. FFAs may also have an impact on insulin production. Long-term FFA exposure reduces beta-cells’ glucose-induced insulin secretion response [7,8]. Liver, muscle cell, and adipocyte sensitivity to insulin is drastically altered during IR; the condition lowers glucose uptake in muscle and fat cells and causes decreased glycogen synthesis and storage and an inability to regulate glucose production in response to insulin in liver cells [5]. 

Reactive oxygen species (ROS) are a group of chemically varied chemicals that can accept or give electrons to biological molecules. In biological systems, ROS synthesis and neutralization are normally in equilibrium with antioxidants and do not cause oxidative damage [9]. In a living organism, an imbalance between prooxidants and antioxidants promotes oxidative stress and cellular damage. Recent research links insulin resistance to oxidative damage [10]. Oxidative stress and diabetes mellitus have complex relationships that exacerbate each other. Oxidative stress reduces beta-cell activity, insulin production, proinsulin membrane inclusion, and glucose-induced exocytosis. It can also trigger pancreatic apoptosis, causing beta-cell death [11,12]. Oxidative stress can reduce glucose transporter 4 (GLUT-4) content by negatively altering gene expression by blocking nuclear factor binding to the insulin responsive region of the GLUT-4 promoter in adipose tissue and skeletal muscle and reducing GLUT-4 translocation to the cell membrane [13]. Oxidative stress can affect insulin signal transduction (IST) by downregulating IST proteins. Free radicals downregulate protein kinase B (Akt), insulin receptor substrate-1 (IRS-1), and glycogen synthase kinase 3 (GSK-3), lowering insulin sensitivity and causing insulin resistance [14]. Furthermore, the nuclear factor erythroid 2 related factor 2 (Nrf2) is a main antioxidant signaling regulator that could prevent the development of metabolic syndrome and related cardiovascular diseases [15,16]. Moreover, newly published data indicate that the redox balance contributes to the effects of Nrf2 on insulin sensitivity and resistance [17]. 

Two major signaling pathways stimulate glucose uptake in skeletal muscle. The insulin-dependent system requires insulin to activate multiple signaling proteins and processes, including IRS-1, phosphoinositide 3-kinase (PI3K), Akt, and GLUT4 translocation, thereby increasing glucose uptake into skeletal muscle [18]. In insulin-stimulated environments, skeletal muscles are major glucose absorption sites and absorb approximately 75% of glucose [4]. GLUT4 facilitates postprandial glucose absorption in skeletal muscle, which is then transported across the plasma membrane (PM) by insulin. A major characteristic of T2DM is decreased GLUT4 intracellular trafficking into the PM [18]. The other major signaling pathway is the insulin-independent route, which is controlled by 5′-adenosine monophosphate-activated protein kinase (AMPK), a heterotrimeric functional enzyme complex regulating energy balance in the cell [19].

*Dillenia indica* (family Dilleniaecae) is commonly known as elephant apple. The fruit pulp is used to treat dandruff and hair loss, while the sepals are used to resolve gastrointestinal issues [20]. *D. indica* exerts anticancer [20], antibacterial, antioxidant [21], analgesic, and anti-inflammatory effects [22]. Methanol and petroleum ether extract from *D. indica* leaves also exert antidiabetic effects and anti-hyperlipidemic properties in alloxan and streptozotocin (STZ)-induced diabetic rats [23,24]. However, the antidiabetic effects of terpenoid-rich *D. indica* bark (TRDI) along with the underlying mechanism remain unclear. To address this knowledge gap, we investigated how TRDI displays antidiabetic potential in PA-induced IR in C2C12 cells and STZ-induced diabetic mice.

## 2. Materials and Methods

### 2.1. Plant Materials, Extraction, and High-Performance Liquid Chromatography (HPLC) 

In August 2019, *D. indica* L. bark from Jahangirnagar University, Bangladesh was taxonomically identified by the National Herbarium of Bangladesh (voucher specimen no. 49403). Dried and coarsely powdered bark (100 g) was extracted three times in 70% ethanol for 12 h at 60 °C. The collected extract was then evaporated under reduced pressure to give viscous ethanolic extract, fractionated for yielding soluble TRDI and insoluble fractions of chloroform, then concentrated by rotating vacuum evaporator (Tokyo Rikakikai Co. Ltd., Tokyo, Japan). Finally, extracts were lyophilized (Ilshinbiobase, Goyang, Korea), dissolved in dimethyl sulfoxide (DMSO), and diluted in Roswell Park Memorial Institute medium to generate a 3 mg/mL stock solution for pharmacological studies. The final DMSO concentration was 0.1%.

Samples (TRDI) were ultrasonically extracted in methanol (1:1; *v*/*v*) and passed through a 0.45 mm filter. A sample (20 µL) was injected onto a HPLC column (XTerraTMRP C18 column, 4.6250 mm length and 5 mm particle size) and eluted in acetonitrile: water (9:1) at pH 3.0 (phosphoric acid) at 1 mL/min, with eluates measured at 210 nm [25]. External standard calibration curves for betulinic acid (BTLA), betulonic acid (BTLNA), and betulin (BTLN) were generated using calibration solutions in the 100–800 mg/L concentration range. Solutions were injected three times, and curves were generated using average areas.

### 2.2. Radical Scavenging Assay

To assess the free radical scavenging capability of TRDI, 2,2-diphenyl-1-picrylhydrazyl (DPPH) and 2,2′-azino-bis (3-ethylbenzothiazoline-6-sulfonic acid (ABTS)-radical scavenging assays were carried out using a previously described protocol [21]. Ascorbic acid was treated as standard antioxidant. The percent inhibition was computed using the following equation:

(1) $$\;Activity\;\left(\%\;inhibition\right)=\left[\frac{\left(Abs_{control}-Abs_{sample}\right)}{Abs_{control}}\right]\;\times \;100$$

where *Abs_control_* is the absorbance of the control sample, and *Abs_sample_* is the absorbance of the experimental sample. All samples were analyzed in triplicate.

### 2.3. α-Glucosidase Inhibition Assay

The inhibitory activity of α-glucosidase was tested using a method by Alam et al. 2018 [1]. Acarbose as a positive control was employed, and the inhibitory percentage was calculated using Equation (1). The kinetic mode of inhibition of BTLA, BTLNA, and BTLN against α-glucosidase was established via a series of sample dilutions where substrate concentrations were changed in the absence or presence of different BTLA, BTLNA, and BTLN doses. BTLA, BTLNA, and BTLN inhibition rates were determined using Lineweaver–Burk plots and identifying the enzyme dissociation constant (Km) and maximum reaction velocity (Vmax).

### 2.4. Cell Culture, Cell Differentiation, and Glucose Uptake Assays

C2C12 cells were cultured in 96-well plates (1 × 10^5^ cells/well) at 37 °C in 5% CO_2_ in Dulbecco’s modified Eagle’s medium (DMEM) plus 10% fetal bovine serum (FBS) and 1% P/S. Once confluent, cells were differentiated for 5 days in DMEM plus 2% horse serum. Then, cells were starved for 12 h in bovine serum-free, low-glucose DMEM.

The colorimetric 3-(4,5-dimethylthiazol-2-yl)-2,5-diphenyl-2H-tetrazolium bromide (MTT) assay was used to measure C2C12 viability. In 96-well plates, 1 × 10^5^ cells/well were cultured for 24 h. After reaching 90% confluence, cells were supplemented with different TRDI concentrations. After 24 h, MTT reagent was added to wells and the plate incubated at 37 °C for 1 h. Intracellular insoluble formazan was then dissolved by adding 100% DMSO, and absorbance was measured at 595 nm in a microplate reader (Perkin Elmer, Wallac Victor, MA, USA). Viability percentages were calculated [1].

### 2.5. Establishing a Palmitic Acid-Induced Insulin Resistance (PA-IR) Model and Glucose Uptake Assay

To develop a PA-IR model, C2C12 myotubes were treated with 250 µM PA (Sigma-Aldrich, St. Louis, MO, USA) for 24 h, as described by Cai et al. [26]. Briefly, PA was dissolved in ethanol and diluted to 1:10 in fatty acid-free (>98%) bovine serum albumin (BSA; Sigma; final concentration = 2% *w*/*v*). Then, PA-BSA was diluted to 1:10 in 1% FBS-DMEM plus 2% (*w*/*v*) BSA.

The 2-(N-(7-nitro-2,1,3-benzoxadiazol-4-yl) amino)-2-deoxyglucopyranoside (2-NBDG) assay was used to determine glucose absorption [1]. Briefly, cells were simultaneously pretreated with recommended TRDI and 250 µM PA doses for 24 h. Cells were washed three times in phosphate buffered saline (PBS) and incubated in the presence or absence of 100 nM insulin for 30 min before 2-NBDG (50 µM) addition for 1 h at 37 °C. Then, cells were rinsed twice in cold PBS and 2-NBDG uptake measured using a fluorometer (Perkin Elmer) at 485 nm and 585 nm.

### 2.6. Intracellular Reactive Oxygen Species (ROS) Measurement 

According to the method described elsewhere, an oxidant-sensitive fluorescent probe 2’,7’-Dichlorofluorescein-diacetate (DCFH-DA) was used to evaluate the intracellular ROS scavenging activity of TRDI [21]. In brief, the cells (1 × 10^5^ cells/mL) were seeded into 96-well plates for 12 h followed by treatment with TRDI (25–75 µg/mL) with or without 250 µM PA (Sigma-Aldrich, St. Louis, MO, USA) for 24 h. The generation of PA-induced ROS as a cellular oxidative stress was measured.

### 2.7. Transfection of Small Interfering RNA (siRNA)

C2C12 cells (1 × 10^5^ cells/mL) were inoculated at a 1 × 10^5^ cells/mL concentration in a 6-well plate for 24 h. Using Lipofectamine RNAiMax (Invitrogen, Carlsbad, CA, USA), the cells were transfected with 10–50 nM siRNA as per manufacturer’s protocol. The si-Nrf2 RNAs and si-Control were obtained from Santa Cruz Biotechnology (catalog number: SC-37049, Santa Cruz, CA, USA).

### 2.8. Protein Extraction and Western Blotting Analysis 

A nuclear and cytoplasmic extraction kit (Sigma-Aldrich Co. St. Louis, MO, USA) was used to extract the nuclear and cytosolic protein. Moreover, cells were washed in cold PBS, and a plasma membrane (PM) protein isolation kit (Invent Biotechnologies, Inc., Eden Prairie, MN, USA) was used to extract PM proteins. According to manufacturer’s instructions, a PM fraction was isolated from cellular components (nuclei, cytosol, and organelles). Bicinchoninic acid (BCA) reagent was used to determine PM fraction protein concentrations.

After treatments, C2C12 myotubes were washed twice in ice-cold PBS and lysed in ice-cold RIPA buffer (Sigma-Aldrich). In the streptozotocin (STZ)-induced mice, at the end of the experiment day we collected the hindlimb skeletal muscle for immunoblotting and histopathological examination. BCA protein assay kit (Pierce, Rockford, IL, USA) was used to determine protein concentrations, after which 35 µg protein was separated using sodium dodecyl sulfate-polyacrylamide gel electrophoresis (10%) and transferred to nitrocellulose membranes (Whatman, Dassel, Germany). Membranes were treated with primary (1:1000) and secondary antibodies (Supplementary Table S1) after blocking in 5% non-fat milk in 1XTris-buffered saline and 0.1% tween (TBST) buffer (1:5000). Protein bands were detected using a SuperSignal West Femto maximum sensitivity substrate (Thermo Fisher Scientific, Rockford, IL, USA) and quantified using Image LabTM Software (Version 5.2.1). Thioredoxin reductase (TrxR) activity colorimetric assay kit (BioVision, catalog No: K763, Waltham, MA, USA) was used to assess TrxR activity according to the manufacturer’s instructions.

### 2.9. The STZ-Induced Diabetes Model

Six-week-old male C57BL/6 mice (Samtako Korea, Osan, Korea) were maintained in a 12 h light/dark cycle and *ad libitum* access to food (lab rodent chow diet which have protein 21.45% and fat 6.24%) and water, in a room maintained at 22 °C ± 10 °C, in 55% ± 5% humidity. Based on Kyungpook National University Committee Laboratory Animal Ethics (KNU 2020-0078, Daegu, Korea) guidelines, individually housed mice were acclimated to the laboratory environment for at least one week before study commencement. 

Mice were divided into four groups (*n* = 6/group):

G1: the control group (animals received normal saline p.o. every odd day for 3 weeks after the STZ-induced diabetic model was established),
G2: the STZ-induced diabetic control group,
G3: the STZ-induced diabetic plus glibenclamide group (animals received glibenclamide at 5 mg/kg body weight p.o. every odd day for 3 weeks after the STZ-induced diabetic model was established), and
G4: the STZ-induced diabetic group plus TRDI (animals received TRDI at 150 mg/kg body weight p.o. every odd day for 3 weeks after the STZ-induced diabetic model was established).

Diabetes was initially induced by administering STZ in a 50 mM citrate buffer (pH 4.5) at 75 mg/kg intraperitoneally for three days in the diabetic groups. A similar citrate buffer volume was given to the G1 control group. Fasting blood glucose levels were assessed at Day 4; mice with levels ≥ 200 mg/dL were chosen for study.

### 2.10. Histochemical Analysis

Mice were humanely sacrificed at study end. Primary organs, including the pancreas, liver, and skeletal muscle, were removed. For western blotting, some skeletal muscle was retained in liquid nitrogen. For histochemistry, the pancreas, liver, and remaining skeletal muscle were embedded in paraffin after preservation in 10% formaldehyde. Hematoxylin and eosin (H&E) staining was used to visualize STZ-induced pancreas-cell shrinkage by microscopy. Periodic acid-Schiff (PAS) staining was used to visualize glycogen levels in skeletal muscle.

### 2.11. Statistical Analysis

Data were analyzed using one-way analysis of variance (ANOVA) and presented as the mean ± standard deviation (SD). Analyses were performed using SPSS for Windows Version 10.07 (SPSS, Chicago, IL, USA), and *p* values < 0.01 or < 0.05 were considered statistically significant.

## 3. Results

### 3.1. High-Performance Liquid Chromatograms (HPLC) Analysis of TRDI Extract

**Figure 1:**
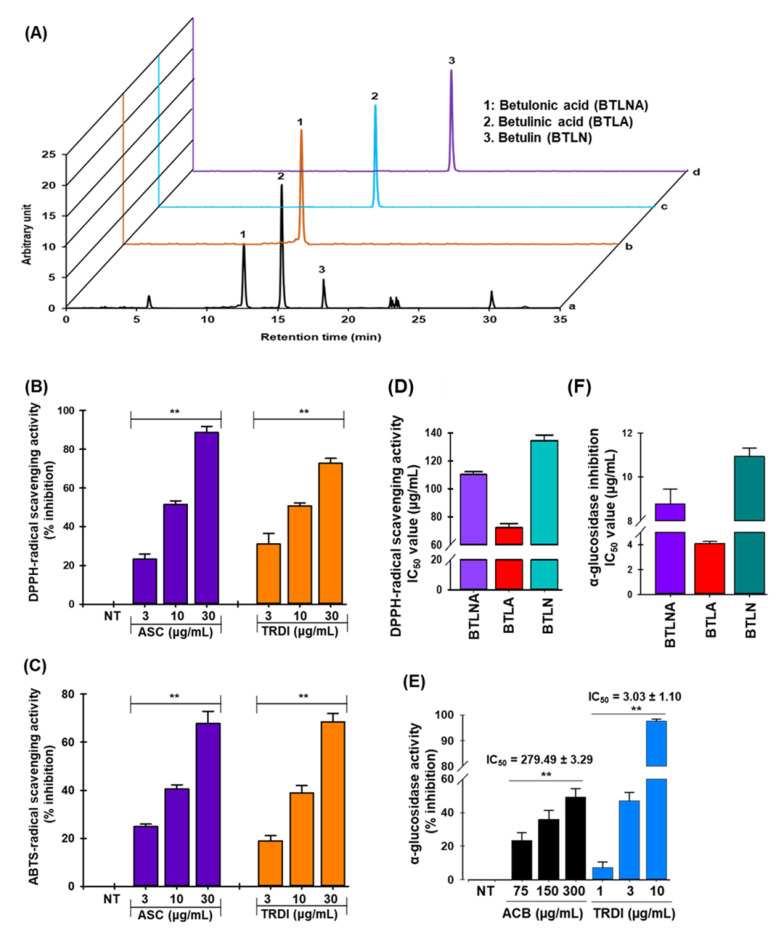
Radical-scavenging and α-glucosidase inhibition activities of terpenoid-rich *D. indica* bark (TRDI) extract: (**A**) high-performance liquid chromatography (HPLC) analysis of TRDI extract; concentration dependent; (**B**) DPPH-; (**C**) ABTS-radical scavenging activities of TRDI; (**D**) DPPH-radical scavenging activity of identified compounds; (**E**) concentration-dependent α-glucosidase inhibitory activity of TRDI; (**F**) a α-glucosidase inhibitory activity of identified terpenoid compounds. Data are presented as the mean ± standard deviation (SD) (*n* = 3). ** *p* < 0.01 compared with the no treatment group. ASC: ascorbic acid; ACB: acarbose; BTLA = betulinic acid; BTLNA = betulonic acid; and BTLN = betulin.

Recent evidence and our previous study indicated that *D. indica* was high in phenolics, flavonoids, and pentacyclic triterpenoids [20,21]. Due to the evidence showing that BTLA showed antioxidant traits, inhibited α-glucosidase, improved insulin sensitivity, and reduced oxidative stress in metabolic syndrome rats [27], we measured this compound (and others) in extracts using HPLC (Figure 1A). TRDI extract contained BTLNA, BTLA, and BTLN at 17.57 mg/g, 29.94 mg/g, and 8.16 mg/g concentrations, respectively (Supplementary Figure S1A–C).

### 3.2. Radical Scavenging Activities of TRDI Extract

The antioxidant activities of phytochemicals involve various molecular mechanisms. Thus, various methods should be used to assess the antioxidant potential of plant extracts [21]. In this study, the antioxidant potential of TRDI extract was analyzed using DPPH-, and ABTS-, radical scavenging assays. As shown in Figure 1B,C, TRDI exhibited a dose-dependent and significant DPPH- and ABTS-radical scavenging potential with IC_50_ values of 9.76 ± 0.50 µg/mL and 17.47 ± 1.31 µg/mL, whereas the positive control, ascorbic acid, had an IC_50_ value of 9.67± 0.47 µg/mL and 17.06 ± 0.48 µg/mL in the DPPH- and ABTS-radical scavenging assay, respectively. These results suggest that TRDI exhibits antioxidant potential through a hydrogen atom transfer and a single electron transfer mechanism. Furthermore, BTLNA, BTLA, and BTLN also had potential to scavenge DPPH-radical scavenging activity in a dose-dependent fashion with IC_50_ value of 112.25 ± 0.58 µg/mL, 75.27 ± 0.67 µg/mL, and 138.29 ± 1.05 µg/mL, respectively (Figure 1D).

### 3.3. TRDI Extract Inhibits α-Glucosidase

TRDI extract substantially suppressed α-glucosidase activity in a concentration-dependent manner (Figure 1E). The IC_50_ was 3.03 ± 1.10 (µg/mL) which was approximately 92-fold greater than acarbose, a competitive α-glucosidase inhibitor (IC_50_ = 279.49 ± 3.29 µg/mL). Next, all three compounds showed concentration-dependent α-glucosidase inhibitory activities with IC_50_ values of 8.25 ± 1.39 µg/mL, 4.27 ± 0.51 µg/mL, and 11.04 ± 1.31 µg/mL, respectively (Figure 1F). Kinetic studies also showed BTLA, BTLNA, and BTLN interactions with α-glucosidase (Supplementary Figure S2A–C); the three compounds were classified as mixed, uncompetitive, and non-competitive inhibitors, respectively. 

### 3.4. Glucose Uptake Facilitated by TRDI in Normal and PA-IR C2C12 Myotubes

**Figure 2:**
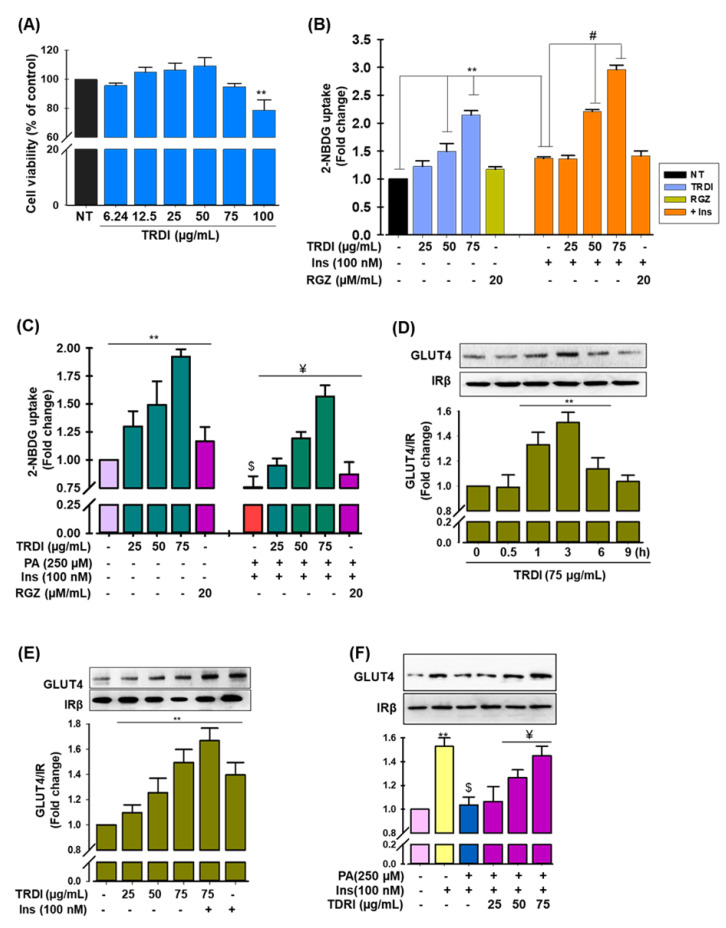
The effect of TRDI extract on glucose uptake and GLUT4 translocation in normal and PA-IR C2C12 cells: (**A**) TRDI cytotoxicity in C2C12 cells. Cells (1 × 10^5^ cells/mL) were treated with different TRDI concentrations (6.24, 12.5, 25, 50, 75, and 100 µg/mL). MTT assays were used to assess TRDI cytotoxicity; (**B**,**C**) A dose-response relationship between the effects of TRDI on glucose uptake in normal and PA-induced C2C12 myotubes was identified. Cells were treated with or without 250 µM PA for 24 h. Then, cells were treated with TRDI alone or with insulin for 30 min in normal and PA-induced cells, and glucose uptake was measured at 3 h. Rosiglitazone (20 μM) was used as a positive control. Data represent the mean ± standard error of the mean (SEM) (*n* = 3). ** *p* < 0 01 versus basal glucose uptake (with insulin stimulation); # *p* < 0.05 versus insulin only activated glucose uptake; ^$^ *p* < 0.05 versus basal glucose uptake in PA-IR C2C12 cells; ^¥^ *p* < 0.05 versus PA-induced treated cells only; (**D**) the time course effect of TRDI extract (75 µg/mL) on GLUT4 translocation to the PM in normal C2C12 cells; (**E**,**F**) the concentration dependent effect of TRDI on GLUT4 translocation to the plasma membrane in normal and PA-induced cells; C2C12 cells were treated with different TRDI concentrations alone for 3 h or with insulin (100 nM) for 30 min. Cell lysates were immunoblotted using the anti-GLUT4 antibody. Experiments were performed in triplicate and the mean ± standard deviation (SD) shown; ** *p* < 0 01 versus basal GLUT4 level; ^$^ *p* < 0.05 versus basal GLUT4 levels in PA-induced cells; ^¥^ *p* < 0.05 versus PA-induced treated cells only; RGZ = rosiglitazone; Ins = insulin; PA = palmitic acid.

TRDI did not show any cellular toxicity up to dose 75 µg/mL in C2C12 cells (Figure 2A). In a non-toxic dose, TRDI activated glucose absorption in C2C12 cells in a dose-dependent manner in both basal and insulin-induced glucose uptake (Figure 2B). Basal and insulin-activated glucose uptake levels increased considerably by 2.14 ± 0.08 and 2.98 ± 0.08-fold, respectively, at the maximum TRDI concentration (75 µg/mL). Rosiglitazone (RGZ), a thiazolidinedione-class antidiabetic drug and used as a positive control in this study, increased basal and insulin-induced glucose absorption by 1.17 ± 0.04 and 1.41 ± 0.09-fold, respectively. As expected, glucose absorption was reduced by 0.25 ± 0.09-fold in C2C12 cells following PA treatment, whereas TRDI administration augmented glucose uptake concentration dependently and was 2.07 ± 0.09-fold higher at the highest TRDI concentration (75 µg/mL) than PA treatment alone (Figure 2C).

### 3.5. TRDI Promotes Glucose Transporter Type 4 (GLUT4) Translocation into the Plasma Membrane (PM) in Normal and PA-IR C2C12 Myotubes

GLUT4 translocation into the PM was enhanced by TRDI administration in a time-dependent manner, peaking at 3 h (Figure 2D). Therefore, this period was chosen for further investigation. TRDI, with or without insulin, stimulated GLUT4 translocation into the PM in a concentration-dependent manner (Figure 2E). Furthermore, immunoblotting showed that after PA treatment, GLUT4 translocation into the PM was dramatically reduced, confirming IR in C2C12 myotubes (Figure 2F). Interestingly, TRDI reversed this in a concentration-dependent manner (Figure 2F).

### 3.6. TRDI Activates Insulin Signaling in Normal and PA-IR C2C12 Myotubes

Glucose uptake via GLUT4 in skeletal muscle is dependent on insulin or AMP-activated kinase (AMPK) signaling pathways [18,19]. To identify the signaling pathways involved in TRDI-mediated glucose uptake, immunoblotting was performed. As shown inFigure 3A, TRDI treatment significantly activated IRS-1, phosphoinositide-dependent kinase-1 (PDK1), and Akt in a time-dependent manner in C2C12 myotubes. Levels peaked at 60 min. To further ascertain if IRS-1 phosphorylation and downstream signaling via TRDI augmented GLUT4 translocation and enhanced glucose uptake, we evaluated the effects of the PI3K inhibitor, LY294002 (LY). 

**Figure 3:**
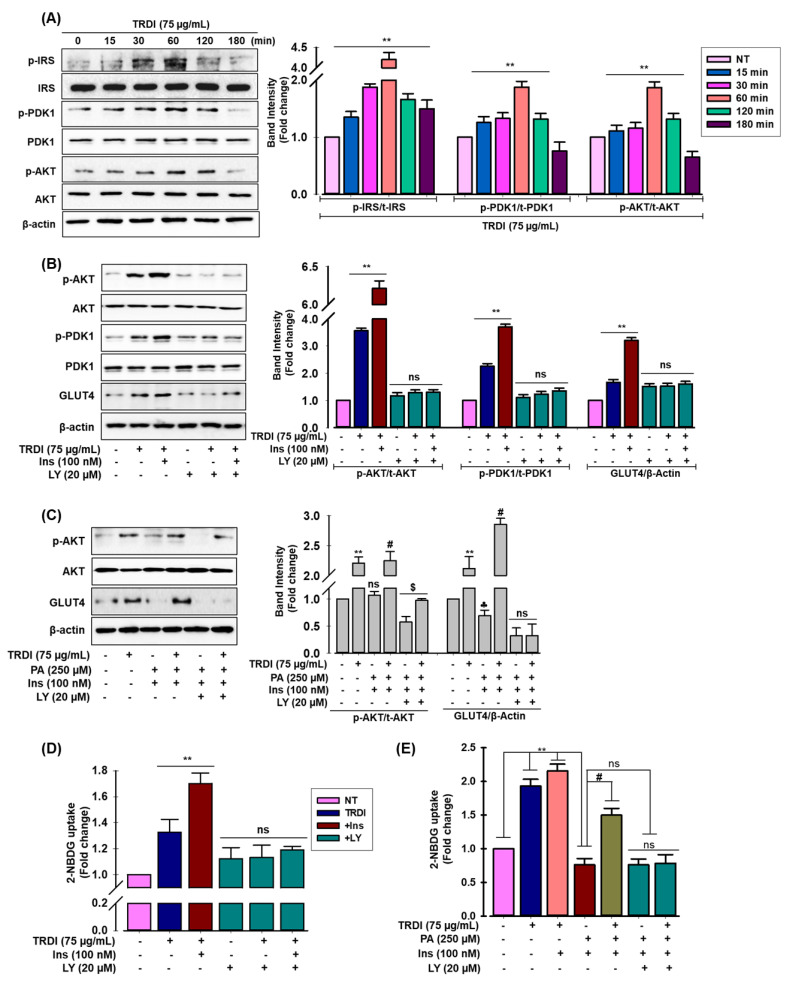
Activation of insulin signaling by TRDI in normal and PA-IR C2C12 cells: (**A**) after serum starvation, TRDI (75 μg/mL) was supplemented to C2C12 myotubes for the indicated times. Cell lysates were immunoblotted to assess IRS-1, PDK1, and Akt phosphorylation levels; relative intensity of phosphorylated protein in adjacent figure. Values are the mean ± standard deviation (SD) from three independent experiments. ** *p* < 0.05 versus basal conditions; (**B**) The PI3K inhibitor (LY294002) was used to treat cells for 30 min before TRDI administration, with or without insulin. Cell lysates were immunoblotted for p-Akt p-PDK1 and GLUT4; relative intensity of phosphorylated proteins in adjacent figure; (**C**) Cells were treated with or without 250 µM PA for 24 h. LY294002 was used to treat cells for 30 min before TRDI administration, with or without insulin. Cell lysates were immunoblotted for p-Akt and GLUT4 expression; relative intensity of phosphorylated proteins in adjacent figure; ** *p* < 0.01 versus basal p-Akt and GLUT4 levels; # *p* < 0.05 versus PA-induced p-Akt; # *p* < 0.05 basal GLUT4 levels in PA-induced C2C12 cells; ^$^ *p* < 0.05 versus basal p-Akt; ^♣^ versus basal GLUT4 levels; (**D**,**E**) Glucose uptake was also measured. Values are the mean ± SD of three independent experiments; ** *p* < 0.01 versus basal glucose uptake; # *p* < 0.05 versus PA-induced glucose uptake; Ns = non-significant.

The LY-treated group downregulated TRDI-stimulated PDK1, Akt, and GLUT4 phosphorylation in the presence and absence of insulin (Figure 3B). Furthermore, the effect of TRDI on PA-IR was confirmed by measuring insulin-signaling-associated proteins in C2C12 cells. PA treatment reduced GLUT4 levels, and Akt phosphorylation had no effect on total GLUT4 or Akt levels, whereas TRDI considerably increased GLUT4 levels and reversed Akt phosphorylation (Figure 3C). Interestingly, basal and insulin-stimulated glucose uptake was also attenuated by LY treatment and abolished TRDI-induced glucose intake (Figure 3D). Similarly, LY administration reduced the effects of TRDI on glucose uptake (Figure 3E) and reversed TRDI-induced GLUT4 expression and Akt phosphorylation (Figure 3C) in PA-induced IR C2C12 myotubes. Thus, by altering Akt/GLUT4 signaling, TRDI improved glucose uptake and insulin sensitivity in PA-induced C2C12 cells.

### 3.7. TRDI Relieves Oxidative Stress in C2C12 Cells via Regulation of Nuclear Factor Erythroid 2 Related Factor 2 (Nrf2) 

**Figure 4:**
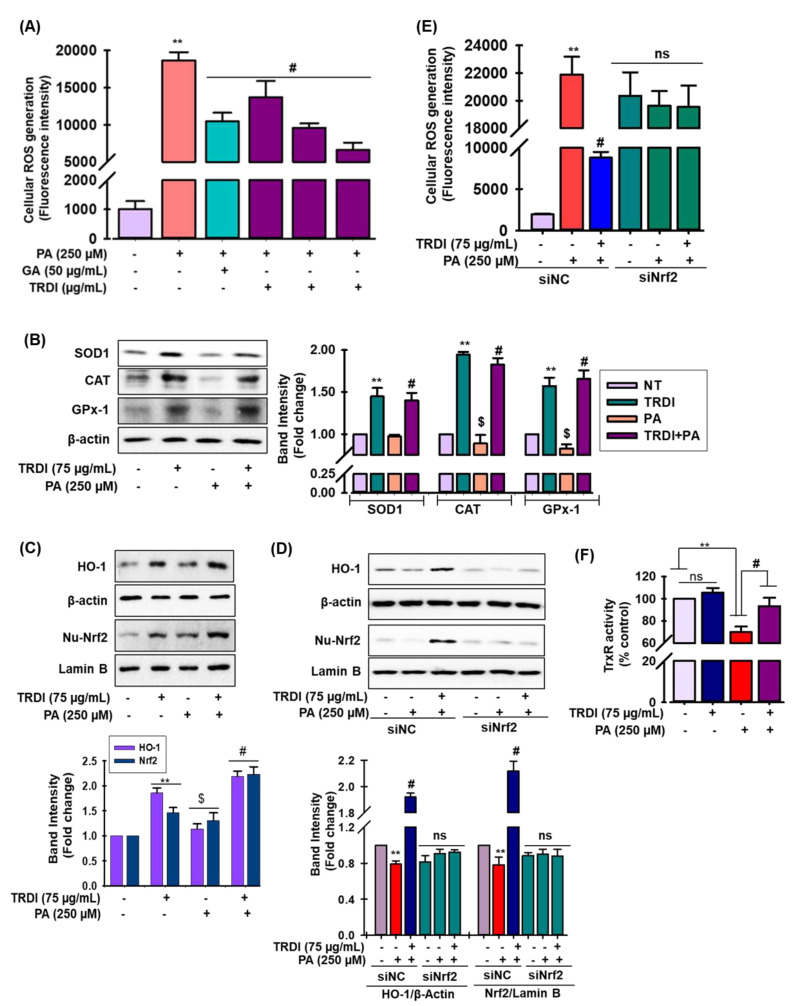
The antioxidant effects of TRDI on PA-induced C2C12 cells: (**A**) cells were pretreated with TRDI and gallic acid for 12 h, followed by 250 µM PA administration and incubation for 24 h. Reactive oxygen species (ROS) was evaluated using the (2,7-dichlorodihydrofluorescein diacetate (DCFH-DA) method; ** *p* < 0.01 versus non-treated cells and # *p* < 0.05 versus PA-induced cells; cell lysates and nuclear fractions were also processed; (**B**) superoxide dismutase 1 (SOD-1), catalase (CAT), glutathione peroxidase 1 (GPx-1) protein expression; (**C**) heme oxygenase-1 (HO-1) and nuclear factor erythroid 2 related factor 2 (Nrf2) levels were quantified using immunoblotting assay; relative protein expression was evaluated using Image Lab Software (version 5.2.1) and expressed in the adjacent figure. (**D**) Cells were treated with TRDI in the presence and absence of PA and small interfering Nrf2 RNA (siNrf2) and HO-1 and Nrf2 levels quantified by immunoblotting; relative protein expression was described in the adjacent figure. Values are the mean ± standard deviation (SD) (*n* = 3); ** *p* < 0.01 versus basal levels; # *p* < 0.05 versus PA-induced levels; ^$^ *p* < 0.01 versus TRDI levels alone. (**E**) Cells were treated with TRDI in the presence and absence of PA and siNrf2, and ROS generation was measured as described in materials and methods; ** *p* < 0.01 versus basal levels; # *p* < 0.05 versus PA-induced levels. (**F**) Thioredoxin reductase (TrxR) activity was assayed in cell lysate; ** *p* < 0.01 versus basal levels; # *p* < 0.05 versus PA-induced levels; siNC = negative control small interfering RNA; ns = non-significant.

ROS formation was significantly increased when PA was supplemented to C2C12 cells (Figure 4A). In a concentration-dependent manner, TRDI pretreatment efficiently suppressed this PA-induced ROS generation (Figure 4A). Also, PA treatment reduced the endogenous levels of the antioxidant enzymes, superoxidase-1 (SOD-1), catalase (CAT), and glutathione peroxidase-1 (GPx-1) in C2C12 cells, whereas TRDI effectively reversed these effects (Figure 4B).

TRDI administration facilitated Nrf2 nuclear translocation and enhanced HO-1 expression in C2C12 cells, but these observations were significantly reduced after PA treatment (Figure 4C). To further validate Nrf2 function in TRDI-induced HO-1 expression, knock down of Nrf2 using small interfering Nrf2 RNA (siNC and siNrf2) experiment was carried out. Transfection of the cells with siNrf2 before TRDI treatment significantly reduced Nrf2 expression (Figure 4D; lower panel), as expected, which eliminated the expression of HO-1 (Figure 4D; upper panel). These results confirm that the TRDI-induced expression of HO-1 is mediated via the Nrf2 pathway. Furthermore, to understand how TRDI impacts the Nrf2 pathway in antioxidant activity, a complementation study was conducted. This study revealed that TRDI-induced ROS scavenging was only partially reduced in the presence of siNrf2 (Figure 4E), indicating that the antioxidant effects of TRDI are only partially enacted via the Nrf2 pathway in PA-stimulated C2C12 cells. The thioredoxin/thioredoxin reductase system is critical for redox signaling and redox environment control in cells [28]. PA treated results dramatically reduced thioredoxin reductase (TrxR) activity in PA-induced C2C12 cells, as seen in Figure 4F, but TRDI administration reversed this activity, although TRDI alone did not boost TrxR activity in contrast to the basal level. 

### 3.8. TRDI Prevents PA-Induced Muscle Cell IR via the Nrf2 Signaling Pathway

**Figure 5:**
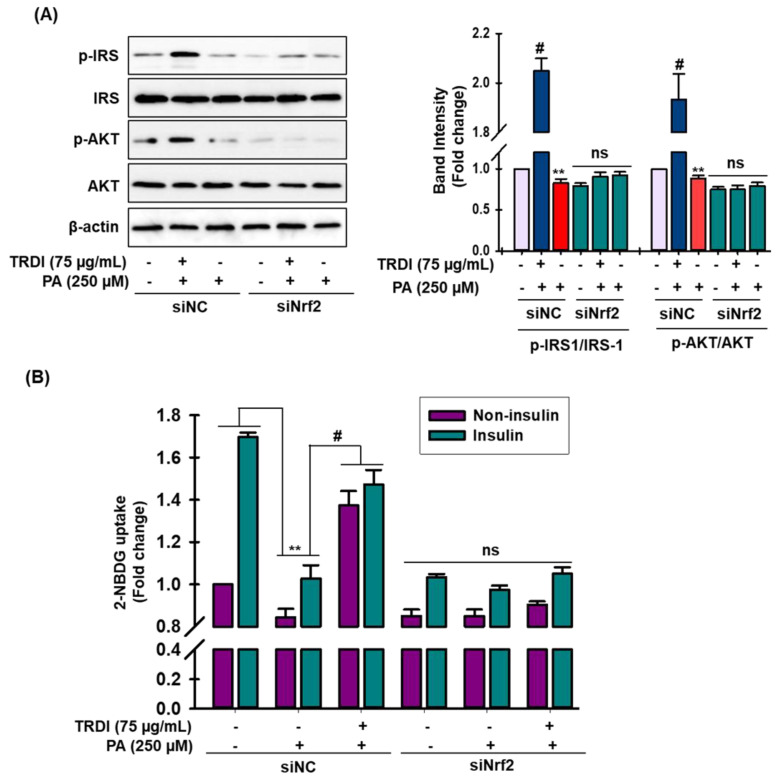
TRDI prevents the activation of Nrf2 signaling in PA-IR treated C2C12 cells: (**A**) cells were treated with or without 250 µM PA for 24 h. Transfection of cells with siNrf2 was carried out for 30 min before TRDI administration. Cell lysates were immunoblotted for p-IRS-1 and p-AKT expression; relative intensity of phosphorylated proteins in adjacent figure; ** *p* < 0.01 versus basal p-IRS-1 and p-AKT levels; # *p* < 0.05 versus PA-induced p-IRS-1 and p-AKT levels; (**B**) transfection of cells with siNrf2 was carried out for 30 min before TRDI administration with or without insulin. Glucose uptake was also measured. Values are the mean ± SD of three independent experiments; ** *p* < 0.01 versus basal glucose uptake; # *p* < 0.05 versus PA-induced glucose uptake; siNC = negative control small interfering RNA; ns = non-significant.

Cumulative studies revealed the role of Nrf2 signaling in the regulation of insulin resistance in smooth muscle [16,29] Hence, immunoblot analysis was performed to validate the role of Nrf2 signaling in the regulation of insulin resistance and glucose uptake in smooth muscle cells. In C2C12 cells, PA treatment significantly attenuated the phosphorylation of IRS-1 and AKT (Figure 5A), while TRDI treatment fully reversed the action of PA (Figure 5A; column 2). Interestingly, in the absence of Nrf2, TRDI completely failed to cause the phosphorylation of IRS-1 and AKT (Figure 5A, column 4–6). Moreover, glucose uptake in normal and insulin-stimulated control cells was markedly attenuated by PA-treated cells (Figure 5B). This could then be significantly counteracted by treatment with TRDI in both conditions. (Figure 5B). However, in the absence of Nrf2, TRDI failed to stimulate the glucose uptake in both conditions, confirming that TRDI failed to resist the effects of IR. 

### 3.9. In Vivo Anti-Diabetic Activity of TRDI in STZ-Stimulated Mice 

**Figure 6:**
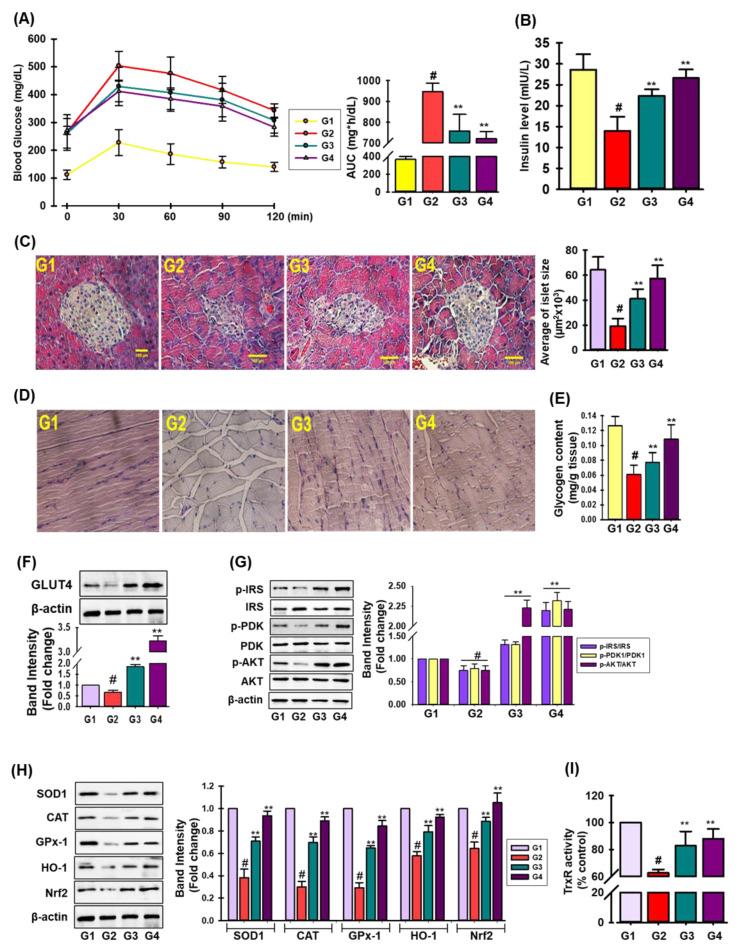
TRDI displays antidiabetic action in STZ-induced diabetes mice: (**A**) oral glucose tolerance effects of TRDI in STZ-induced diabetes mice; fasting mice were orally treated with the G1 group (control, *n* = 6), G2 (STZ control group, *n* = 6), G3 (STZ+glibenclamide, 5 mg/kg, *n* = 6) and G4 (STZ+TRDI, 150 mg/kg, *n* = 6), and an oral glucose tolerance test was monitored after an oral load of glucose (1.0 g/kg) at various time intervals. The AUC is shown in the right panel; (**B**) after treatment, blood insulin level; (**C**) hematoxylin and eosin staining of the pancreas in STZ-induced diabetic mice (Scale bar = 100 μm); the adjacent bar diagram represents average islet size using Image J software (version 1.52); (**D**) representative images of periodic acid-Schiff-stained skeletal muscle in STZ-induced diabetes mice (scale bar = 100 μm); (**E**) glycogen content in skeletal muscle in STZ-induced diabetes mice; (**F**) after treatments, skeletal muscle was assessed for GLUT4 levels by immunoblotting. Statistical data are shown in the adjacent figure; (**G**) skeletal tissue underwent immunoblotting to determine insulin signaling biomarkers. Gel images represent three independent experiments, and relative protein phosphorylation levels are shown in the right panel; (**H**) expression of antioxidant biomarker in skeletal muscle in STZ-treated diabetes mice; # *p* < 0.01 versus the G1 group; ** *p* < 0.05 versus the STZ-induced only group (G2); (**I**) TrxR activity in skeletal muscle in STZ-treated diabetes mice; # *p* < 0.01 versus the G1 group; ** *p* < 0.05 versus the STZ-induced only group (G2).

Due to the high glucose absorption capacity of TRDI via the stimulation of insulin signaling pathways in PA-IR C2C12 cells, we evaluated TRDI hypoglycemic effects in STZ-induced diabetic mice. The OGTT assay was used to determine the anti-hyperglycemic efficacy of hypoglycemic medication [1]. As shown in Figure 6A, the TRDI treatment group (G4) displayed significantly lower blood glucose levels than the positive control, the glibenclamide treatment group (G3) (Supplementary Figure S3). In STZ-induced diabetic model, the plasma insulin level in the STZ-induced diabatic control group (G2) was significantly lower than that of the normal control group (G1), whereas the TRDI treatment group (G4) significantly increased the plasma insulin level (Figure 6B). As shown (Figure 6C), diabetic control animals (G2) exhibited extensive islet shrinkage and lower numbers of islet cells in the pancreas when compared with control animals (G1), whereas G4 animals (TRDI; 150 mg/kg) exhibited islet cells, normal acini cells, and few pancreatic cell regeneration foci. As excess glucose is stored as glycogen in the liver and skeletal muscle [30], we examined glycogen levels in skeletal muscle in the STZ-induced diabetic mice. As indicated (Figure 6D,E), glycogen levels in the skeletal muscle had increased significantly in G4 when compared with G2 and G3. TRDI (G4) displayed significantly augmented GLUT4 levels in skeletal muscle (Figure 6F). Additionally, we investigated if TRDI elevated insulin signaling in these mice; IRS-1, PDK-1, and Akt (Figure 6G) were phosphorylated in the STZ-induced diabetes plus TRDI group (G4). In animal models, an STZ treatment downregulated the protein expression of SOD1, CAT, GPx-1, HO-1 and Nrf2 (Figure 6H), while TRDI treatment significantly reversed this effect. The decreased Nrf2 expression suggests that a STZ treatment inhibits the Nrf2 signaling pathway. Furthermore, an STZ insult significantly reduced the TrxR activity when compared to the control group (G1), while both glibenclamide- and TRDI-treated group (G3 and G4, respectively) reverse this effect significantly (Figure 6I). These data corroborated in vitro findings and suggested TRDI modulated blood glucose levels in STZ-induced diabetic mice by increasing insulin signaling, mitigating oxidative stress by the induction of primary antioxidant enzymes, and resulting Nrf2 signaling in skeletal muscle.

## 4. Discussion

DM is a multifaceted metabolic condition with several negative health implications. To combat this, extensive methods and therapeutics are used to improve glycemic control such as insulin sensitizers, insulin secretagogues, and prandial glucose regulators [30]. However, some drugs elicit serious side effects in patients with poor tolerance. Thus, natural products and pharmaceuticals must be comprehensively investigated and developed to treat DM with minimal side effects [31]. While considerable evidence suggests *D. indica* leaves elicit antioxidant and antidiabetic properties in alloxan and STZ-induced diabetic models [23,24], the underlying mechanisms remain unknown. Additionally, *D. indica* bark exhibits antioxidant activity via Nrf2/HO-1 signaling activation [21]. Thus, our goals were two-fold: to determine if a triterpenoid fraction of *D. indica* bark exerted antidiabetic effects in PA-induced C2C12 cells and STZ-induced diabetic mice and to identify these mechanisms.

In cells, α-Glucosidase catalyzes the dissociation of 1, 4 glycosidic bonds from the non-reducing ends of oligosaccharides to produce α-glucose which enters the bloodstream via the small intestine. It is considered a good model for studying the effects of nutraceuticals on DM [32]. Thus, blocking α-glucosidase could lower blood sugar levels by preventing α-glucose production. We showed that TRDI and associated pentacyclic triterpenoids substantially and dose-dependently inhibited α-glucosidase activity. Our findings agreed with previous studies showing that BTLA, BTLNA, and BTLN were effective α-glucosidase inhibitors. Similarly, lupan-type triterpenes were also shown to inhibit α-glucosidase [33]. Structure–activity relationship studies revealed that hydroxyl group at position C-3 and the carboxylic acid group at position C-17 in lupan-type triterpenoid play a key role in α-glucosidase inhibition activity [34]. Furthermore, 2,3-seco-20(29)-Lupene-2,3-dioic acid from *Fagus hayatae* also demonstrated good inhibitory effect against -glucosidase [35]. These findings imply that TRDI, as well as lupan-type triterpenoids, could be a potential hypoglycemic drug for the treatment of diabetes. However, whether triterpenoids can block -glucosidase in vivo is still unknown.

IR occurs when insulin-targeted tissues do not respond to insulin stimulation. In the physiological state, insulin receptor binding induces IRS-1 phosphorylation which stimulates GLUT4 and glucose transport upon insulin signal transduction. IRS-1/GLUT4 signaling is suppressed during IR. Insulin or muscular contractions translocate GLUT4 from cytoplasmic GLUT4 vesicles to the PM, where they have key glucose transport roles into the cell. IR in T2DM is caused by decreased GLUT4 translocation from GLUT4 vesicles to the PM [36]. In our study, we observed that TRDI elevated glucose uptake in both normal and IR C2C12 cells by enhancing GLUT4 translocation into the PM (Figure 2). TRDI also increased IRS-1, PDK1, and Akt phosphorylation (Figure 3A). In cultured C2C12 skeletal muscle cells, betulinic acid boosted glucose uptake, and the terpenoid-rich fraction of *Cleome droserifolia* increased baseline glucose uptake [37]. In addition, a novel pentacyclic triterpenoid isolated from the stem bark of *Sorbus decora*, 23, 28-dihydroxylupan-20(29)-ene- 3β caffeate, was found to increase glucose absorption in C2C12 cells [38]. Furthermore, an Akt inhibitor was used to investigate if TRDI increased glucose absorption by activating Akt signaling; it substantially decreased Akt phosphorylation and GLUT4 translocation (Figure 3B). Inhibitor administration also significantly reduced basal and insulin-stimulated glucose uptake, suggesting IRS-1 downstream signals and Akt were implicated in TRDI-induced insulin signaling. 

Fatty acids are major cell membrane components and are involved in several physiological processes, including energy storage, membrane deposition, intracellular signaling, and gene transcriptional regulation [39]. However, a chronic elevation in plasma free saturated fatty acids (FSFAs) have critical roles during IR and T2DM pathophysiology [5]. FSFAs are also implicated in the relationship between IR and β-cell dysfunction. Elevated PA is frequently identified during IR, and it also stimulates ROS [40]. Here, we generated an IR model in C2C12 myotubes using 250 μM PA. We showed that insulin infusion alone increased glucose uptake and that TRDI dose dependently elevated insulin-stimulated glucose uptake (Figure 2B,C). Akt is required for several molecular functions, including glucose metabolism, cell proliferation, transcription, and cell migration [41]. We showed that TRDI inhibited a PA-induced decrease in glucose uptake, p-Akt, GLUT4 expression (Figure 3C), and glucose levels (Figure 3E) in C2C12 myotubes. By blocking the transcription factor linked with the activation of genes involved in the manufacture of cholesterol, fatty acid, and triglyceride, BTLN has shown a considerable reduction in blood glucose levels when compared to normal glibenclamide, resulting in decreased insulin resistance [42]. In STZ-nicotinamide-induced diabetic mice, BTLA decreased blood glucose and improved insulin sensitivity [27].

Oxidative stress is induced by excessive ROS and is a common pathogenic component in islet cell death, IR, and T2DM [40]. While physiological ROS levels influence intermediates in the insulin signaling cascade, excessive ROS produced during food excess induces oxidative damage prior to IR, both in vivo and in vitro [13,16]. However, the antioxidant enzymes, SOD, Gpx-1, CAT, and TrxR scavenge free radicals and prevent oxidative damage [41]. So, antioxidant substances that reduce oxidative stress are therefore beneficial in the treatment of diabetes. TRDI exerted a substantial anti-oxidative impact in PA-induced C2C12 cells by reducing ROS and increasing SOD, CAT, Gpx-1, and TrxR levels. *D. indica* bark extracts were previously reported to scavenge/inhibit ROS formation [21]. Lupeol, a triterpene present in mango, has been proven to be effective in reducing oxidative stress, lowering ROS levels, and restoring the antioxidant defense system in the liver of Swiss albino mice [43]. It also plays an important role in diabetes treatment. Total terpenes from *Fructus corni* (TFC) reduced cardiac problems in alloxan-induced diabetic rats by reducing oxidative stress, boosting SOD, and lowering blood glucose levels [44]. The antioxidant defense system of STZ-induced diabetic rats was additionally protected by the natural monocyclic monoterpene D-limonene, which is found primarily in the essential oil of citrus fruits [45].

Nrf2 is a key inducible transcription factor safeguarding redox homeostasis during oxidative stress [21]. The protein interacts with antioxidant response elements to regulate the expression of downstream genes (e.g., *HO-1*), which encode detoxifying and antioxidant enzymes and other proteins [21]. Thus, excess ROS is scavenged by Nrf2 activation. Furthermore, natural products with antioxidant abilities can stimulate HO-1 production and manifest their therapeutic effects via the HO-1-dependent pathway [21,46]. *D. indica* bark was previously shown to induce HO-1 production by activating Nrf2 in RAW264.7 cells [21]. Thus, we investigated if HO-1 expression contributed to the antioxidant effects of TRDI under our study conditions. In PA-induced C2C12 cells, TRDI administration increased Nrf2 nuclear translocation and increased HO-1 expression (Figure 4D). Furthermore, absence of Nrf2 inhibited this response and suggested that HO-1 production by TRDI was mediated by Nrf2 activation (Figure 4D). Loboda et al. [47] revealed that BTLN binds in ARE sequence resulting in the activation of Nrf2 in endothelial cells. 

STZ causes diabetes by inducing pancreatic cell cytotoxicity, resulting in hyperglycemia and oxidative stress [23]. We showed that TRDI reduced the harmful effects of STZ toward pancreatic β-cells (Figure 6C), consistent with Kaur et al. [48] who showed that *D. indica* leaves and a chromane compound significantly reduced cytotoxicity in cells subjected to STZ. Furthermore, when compared to metformin, BTLA had a greater effect on pancreatic histology and IR. It was discovered that betulinic acid increased glucose absorption and increased glycogen production. A steroid nucleus is thought to be necessary for a high hypoglycemia effect and perhaps conditioning the high degree of lipophilicity, as well as having antidiabetic effects with minimal toxicity [49]. The mechanism of action of terpenoids and steroids in in vivo studies, according to Li et al. [50], may be due to stimulation of pancreatic islets, which leads to an increase in insulin-induced glucose absorption. Additionally, and pharmacodynamically, if a pharmacological substance exerts activity in certain tissues, signaling is stimulated in these tissues. Our findings showed that TRDI was a potent insulin mimic in skeletal muscle and lowered blood glucose levels (Figure 6A). Bassic acid, an unsaturated triterpene isolated from an ethanol extract of *Bumelia sartorum* root bark, increased hypoglycemic action by increasing insulin release from pancreatic cells [51]. Additionally, TRDI (G4 group) increased GLUT4 expression and glycogen levels in the skeletal muscle in diabetic mice. Also, a single TRDI dose (G4) increased IRS-1, PDK1, and Akt phosphorylation in diabetic mice (Figure 6F) when compared with the STZ-induced diabetes control group (G2), supporting our in vitro and in vivo findings. In addition, TRDI treatment augmented the primary antioxidant protein expression, TrxR activity as well as HO-1 and Nrf2 in skeletal muscle. Thus, TRDI displayed considerable hypoglycemic effects when compared with a hypoglycemic drug in STZ-induced diabetic mice. Tetracyclic triterpenes were found to drastically lower blood glucose levels in mice. In STZ-induced diabetic mice, a terpenoid-rich fraction extracted from *H. coronarium* rhizomes significantly lowered blood glucose levels and increased antioxidant enzyme activity, resulting in normal blood glucose levels. In IR cell lines, this component also improved glucose intake [52]. 

## 5. Conclusions

**Figure 7:**
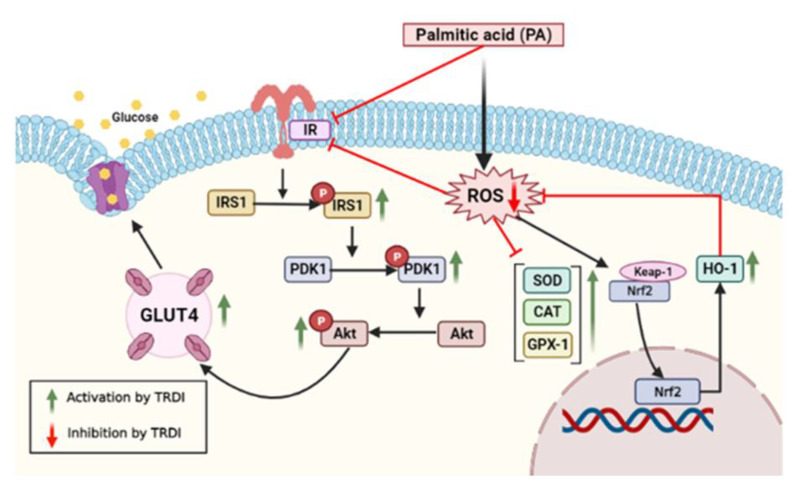
A proposed mechanism for TRDI during glucose homeostasis.

TRDI competitively inhibited α-glucosidase activity in a dose-dependent manner. Additionally, it enhanced GLUT4 translocation and activated the insulin signaling pathway (e.g., IRS-1 and Akt signaling) in basal and PA-IR C2C12 myotubes (Figure 7). Ours is the first study to show that a triterpenoid fraction of *D. indica* L. bark elevated both baseline and insulin-stimulated glucose absorption by simulating insulin signaling and mitigating oxidative stress through regulation of Nrf2 signaling and improving the insulin resistance in skeletal muscle. Our innovative framework may help formulate novel glucose management therapeutics in the future using *D. indica* bark. 

## Data Availability

The data presented in this study are openly available.

## Supplementary Materials

The following supporting information can be downloaded at: https://www.mdpi.com/article/10.3390/antiox11071227/s1, Figure S1: Quantification of pentacyclic triterpenoids presence in *D. indica* bark by HPLC analysis; Figure S2: Enzyme kinetic analysis of identified compounds of TRDI; Figure S3: The effects of TRDI on blood glucose level on STZ-induced diabetic mice; Table S1: List of antibodies used in this study; Table S2: Enzyme kinetic analysis of identified compounds of TRDI.
