# Supplementary material for: Terpenoid-Rich Extract of Dillenia indica L. Bark Displays Antidiabetic Action in Insulin-Resistant C2C12 Cells and STZ-Induced Diabetic Mice by Attenuation of Oxidative Stress
Source: Antioxidants (Basel). 2022 Jun 23;11(7):1227. doi: 10.3390/antiox11071227 (PMC9312268; doi:10.3390/antiox11071227)
Supplement: Supplementary file 1 [file antioxidants-11-01227-s001.zip › antioxidants-1748688-supplementary.pdf]

Supplementary data set

Terpenoid rich extract of *Dillenia indica* L. bark display antidiabetic action in insulin resistant C2C12 cells and STZ-induced diabetic mice by attenuation of oxidative stress

Bo-Rim Song[1], Md Badrul Alam[1,2,] Sang-Han Lee[1,2**]

[a]Department of Food Science and Biotechnology, Graduate School, Kyungpook National University, Daegu 41566, Korea

[b]Food and Bio-Industry Research Institute, Inner Beauty/Antiaging Center, Kyungpook National University, Daegu 41566, Korea

*Corresponding author:

Dr. Sang-Han Lee; Department of Food Science and Biotechnology, Kyungpook National University, Daegu 41566, Korea, Phone: (82)053-950-7754 (Office); (82)010-2537-7659 (Mobile); Fax: 053-950-6772; Email: sang@knu.ac.kr

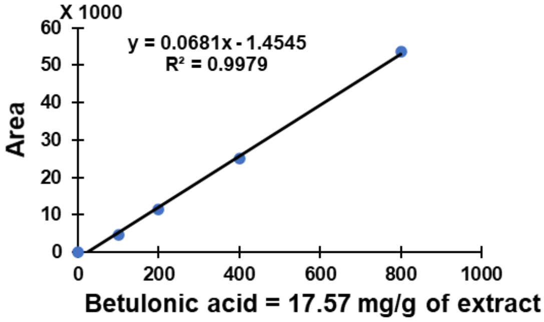

(a)

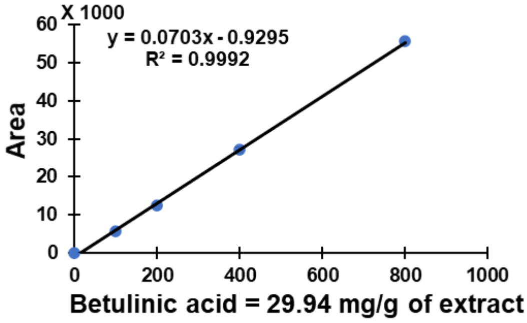

(b)

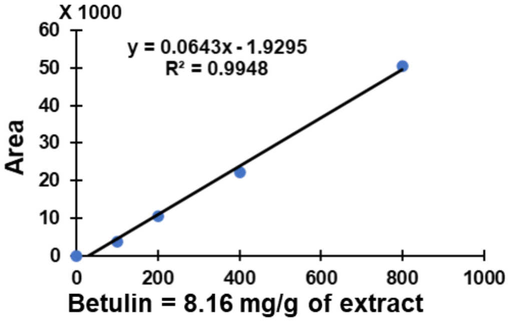

(c)

Figure S1: Quantification of pentacyclic triterpenoids presence in *D. indica* bark by HPLC analysis. Betulonic acid (a); betulinic acid (b) and botulin (c).

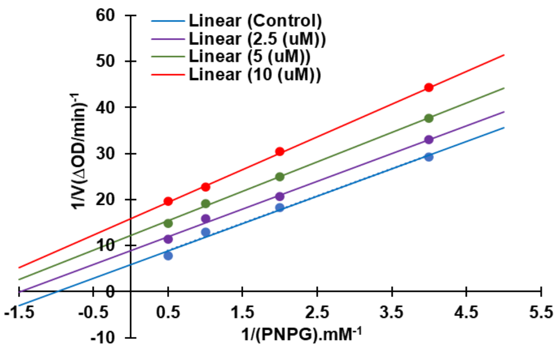

(a)

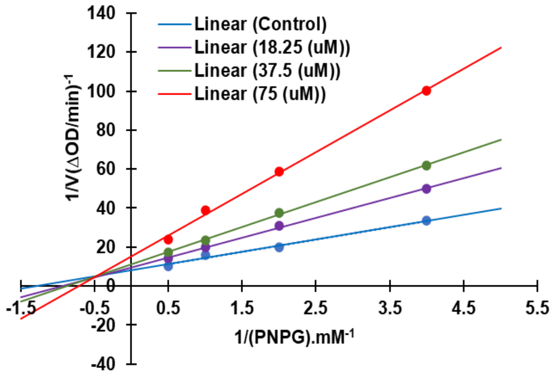

(b)

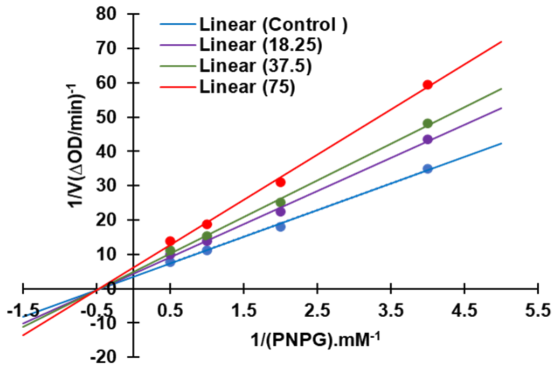

(c)

Figure S2: Enzyme kinetic analysis of identified compounds of TRDI by creating a Lineweaver–Burk plot using 1/substrate concentration vs 1/absorbance change per min. Betulonic acid (a); betulinic acid (b) and botulin (c).

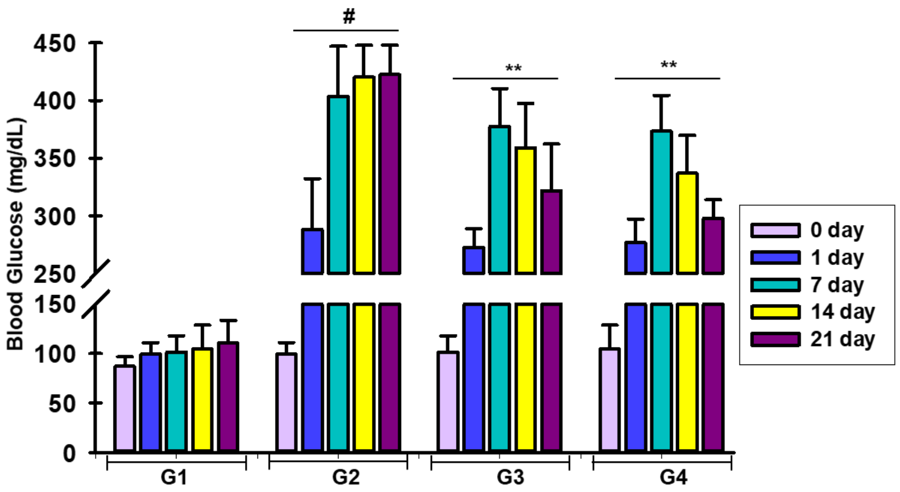

Figure S3: The effects of TRDI on blood glucose level on STZ-induced diabetic mice. The blood glucose level was checked before STZ induction (day 0). After STZ induction, mice were treated with TRDI (150 mg/kg) and glibenclamide (5 mg/kg) p.o. for 3 week. Blood glucose level was checked at every week.

Table S1: List of antibodies used in this study

Antibody	Dilution	Supplier	Catalog No.	Host	MW, kDa
Anti-GLUT4	1:1000	Cell Signaling Technology, Danvers, MA, USA	2213	Mouse	50
Anti-IRβ	1:1000	Cell Signaling Technology, Danvers, MA, USA	3020	Mouse	95
Anti-p-IRS-1	1:1000	Cell Signaling Technology, Danvers, MA, USA	3193	Mouse	180
Anti-IRS-1	1:1000	Cell Signaling Technology, Danvers, MA, USA	3194	Mouse	180
Anti-p-Akt	1:1000	Cell Signaling Technology, Danvers, MA, USA	9271	Rabbit	60
Anti-Akt	1:1000	Cell Signaling Technology, Danvers, MA, USA	4685	Rabbit	60
Anti-p-PDK1	1:1000	Cell Signaling Technology, Danvers, MA, USA	3061	Rabbit	58-68
Anti-PDK1	1:1000	Cell Signaling Technology, Danvers, MA, USA	5662	Rabbit	58-68
Anti-SOD1	1:1000	Bioworld Technology, Inc.	BS91268	Rabbit	23
Anti-catalase	1:1000	Bioworld Technology, Inc.	BS90194	Rabbit	68
anti-GPx-1	1:1000	Bioworld Technology, Inc.	MB9027	Mouse	92
Anti-HO-1	1:1000	Santa Cruz Biotechnology, Inc.	sc-136256	Mouse	32
Anti Nrf2	1:1000	Santa Cruz Biotechnology, Inc.	sc-81342	Mouse	66
Anti-Lamin B	1:1000	Bioworld Technology, Inc.	BS3547	Rabbit	66
Anti-β-actin	1:1000	Santa Cruz Biotechnology, Santa Cruz, CA, USA	Sc-47778	Mouse	43

Table S2: Enzyme kinetic analysis of identified compounds of TRDI

Compound	Concentration (mM)	Km (mM)	Vmax (ΔOD/min)	Mode of inhibition
Betulin	NT	2.2 x10^{-3}	29.21 x10^{-3}	Non-competitive
	18.25 x10^{-3}	2.2 x10^{-3}	22.62 x10^{-3}	
	37.5 x10^{-3}	2.2 x10^{-3}	20.34 x10^{-3}	
	75 x10^{-3}	2.2 x10^{-3}	16.21 x10^{-3}	
Betulinic acid	NT	1.0 x10^{-3}	12.41 x10^{-3}	Mixed
	18.25 x10^{-3}	1.1 x10^{-3}	10.35 x10^{-3}	
	37.5 x10^{-3}	1.2 x10^{-3}	8.96 x10^{-3}	
	75 x10^{-3}	1.3 x10^{-3}	6.53 x10^{-3}	
Betulonic acid	NT	1.00 x10^{-3}	16.87 x10^{-3}	Uncompetitive
	2.5 x10^{-3}	0.68 x10^{-3}	11.24 x10^{-3}	
	5 x10^{-3}	0.53 x10^{-3}	8.22 x10^{-3}	
	10 x10^{-3}	0.45 x10^{-3}	6.28 x10^{-3}	
